# Exploring the role of NCCR variation on JC polyomavirus expression from dual reporter minicircles

**DOI:** 10.1371/journal.pone.0199171

**Published:** 2018-06-26

**Authors:** Anne-Sophie L’Honneur, Hervé Leh, Fanny Laurent-Tchenio, Uriel Hazan, Flore Rozenberg, Stéphanie Bury-Moné

**Affiliations:** 1 Université Paris Descartes, INSERM Paris, France; 2 Assistance Publique—Hôpitaux de Paris, Hôpital Cochin, Service de Virologie, Paris, France; 3 LBPA, Université Paris Saclay, CNRS, ENS Paris Saclay, Cachan, France; 4 Institute for Integrative Biology of the Cell (I2BC), CEA, CNRS, Université Paris-Sud, Université Paris-Saclay, Gif-Sur-Yvette, France; Institut Pasteur, FRANCE

## Abstract

JC virus (JCV), a ubiquitous human polyomavirus, can cause fatal progressive multifocal leukoencephalopathy (PML) in immune compromised patients. The viral genome is composed of two conserved coding regions separated by a highly variable non-coding control region (NCCR). We analyzed the NCCR sequence from 10 PML JCV strains and found new mutations. Remarkably, the NCCR *f* section was mutated in most cases. We therefore explored the importance of this section in JCV expression in renal (HEK293H) and glioblastoma (U-87MG) cell lines, by adapting the emerging technology of DNA minicircles. Using bidirectional fluorescent reporters, we revealed that impaired NCCR-driven late expression in glioblastoma cells was restored by a short deletion overlapping *e* and *f* sections. This study evidenced a relevant link between JCV NCCR polymorphism and cell-type dependent expression. The use of DNA minicircles opens new insights for monitoring the impact of NCCR variation.

## Introduction

Progressive multifocal leukoencephalopathy (PML) is an uncommon and severe demyelinating disease highly associated with underlying immune defects. Sporadic PML cases were primarily described in patients with B cell malignancies, mainly Hodgkin’s disease and chronic lymphocytic leukemia [1, 2]. The incidence of PML sharply increased in the 1980s, when human immunodeficiency virus (HIV) infection/AIDS was identified as a major risk factor for PML [3, 4], later significantly reduced by highly active antiretroviral therapy (HAART) [5]. More recently, immunomodulatory therapies such as the α-4-β-1 and α-4-β-7-anti-integrin, Natalizumab, in multiple sclerosis patients, were shown to carry a novel PML-inducing risk [6–8], raising new interest in the comprehension of the disease.

PML is caused by lytic infection of oligodendrocytes by JC virus (JCV), the first human member of the *Polyomaviridae* family [9] initially isolated from PML brain lesions in cultured human fetal glial cells [10]. JCV infection is highly prevalent in human population and mostly asymptomatic [11]. The virus persists lifelong in its host, either in a latent or low productive state in kidney, and is intermittently excreted in urine [12, 13]. JCV genome has also been detected in lymph nodes [14, 15] and bone marrow [15–17] which may be sites of latency, and in tonsils [18–20] which are thought to be the site of primary infection. How and when JCV gains access to the brain remains unknown.

**Fig 1:**
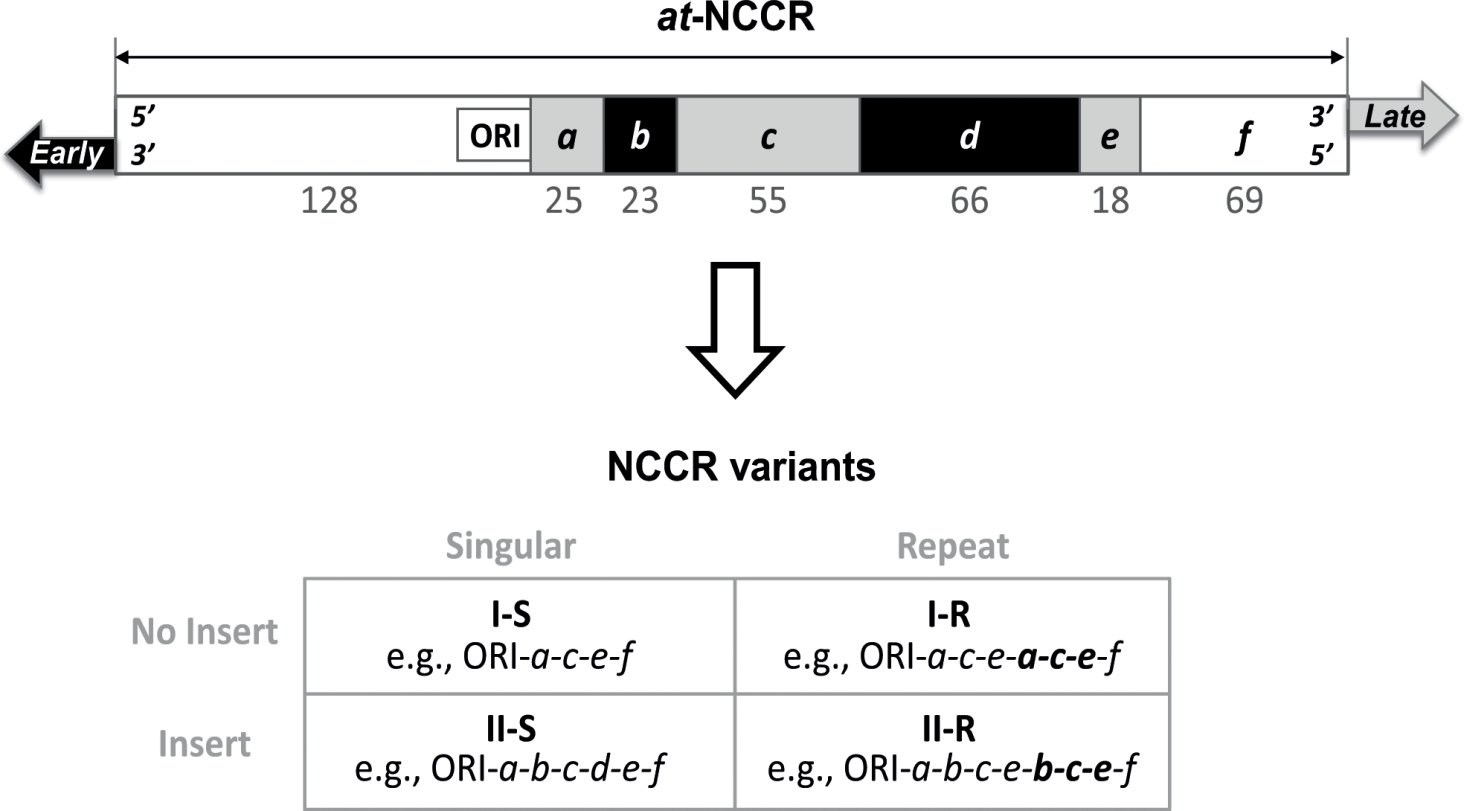
Schematic representation of the JCV NCCRs. The NCCR is delimited by the translation start sites of early and late regions. A highly conserved region which includes ORI is followed by sections *a*, *b*, *c*, *d*, *e* and *f* in the archetype *at*-NCCR. These regions have been defined because of their deletion and/or repeat in variant NCCRs. Accordingly, NCCRs have been classified into four groups [25]: I-S, I-R (like Mad-1 and Mad4), II-S and II-R (like Mad-7 and Mad-8). NCCRs of type I do not contain insert whereas NCCRs of type II present an insertion of at least a portion of the sequence from one of the sections *b* or *d*. The sub-types “S” (for singular) do not present a repeat whereas the subtypes “R” (for repeat) do. NCCRs of type II-S are also named archetype or archetype-like if deletions occur. NCCRs of type II-R have generally been termed rearranged NCCRs (“*rr*-NCCR”) [25]. Numbers indicate the base-pair length of each section within CY *at*-NCCR [12].

The viral particle is composed of a non-enveloped icosahedral capsid containing a circular supercoiled double-stranded DNA genome (≈ 5 Kb). JCV genome is composed of early and late genes that are transcribed in opposite directions from opposite strands of DNA, separated by a non-coding control region (NCCR). As described for other polyomaviruses such as Simian Virus 40 (SV40) and BK polyomavirus (BKV) [21–23], early transcription leads to expression of T antigens required to activate viral replication. Late transcription results in expression of a regulatory protein named agnoprotein and of structural viral proteins leading to production of neosynthesized viral particles. The NCCR is a key regulatory region (≈ 400 bp) harboring both sequences required for replication (ORI, the origin of viral replication) and for transcription (several promoters and *cis*-regulating elements). Most urine-derived JCV NCCR sequences, named ‘archetype‘ (*at*-NCCR), present an initial regulatory region containing the ORI followed by 6 distinct successive sections named *a* to *f* [12, 24, 25] (**Fig 1**). Because of their excretion in urine [26–29] and detection in urban sewage [30], archetype viruses are considered the source of inter-individual oropharyngeal transmission [11].

In contrast, the first described JCV sequence [10], which was obtained from PML lesions (named Mad-1 and later ‘prototype’), was characterized by the absence of *b* and *d* sections and duplication of the *a-c-e* sequence [31], suggesting retrospectively that prototype virus derives by deletion from archetype virus. Analysis of numerous PML NCCR sequences has shown their great variability with repeats, deletions, and point mutations [24, 31–37] (**Fig 1**). In addition to the inter-compartment variability within patients, wide intra-compartment variability has been observed [34, 36, 38–44]. These rearranged NCCRs (*rr*-NCCR) present a modified pattern of binding sites for cell-specific transcription factors, thus highly suggesting that they could play a major role in cell-type specificity, and JCV replication within the brain [11]. The precise nature of the link between NCCR variation and JCV tropism remains to be established.

Several studies attempted to explore this issue, and developed assays based on bioluminescent or radioactive detection of reporter gene expression from unidirectional reporters [45–47], and more recently based on viral replication [37], RNA quantification [48] and/or bidirectional fluorescent reporter genes analysis by microscopy [37]. In this study, we analyzed JCV NCCR sequences from PML strains and set up a new genetic tool to easily monitor expression from *rr*- and *at*- NCCRs by flow cytometry. Since the *f* section contained mutations in most cases, we specifically explored the impact of a naturally occurring deletion within this region on JCV expression, in relevant renal and glial cell lines.

## Materials and methods

### Patients and samples

Viral DNA recovered from cerebrospinal fluid (CSF), cerebral biopsy (CB) and urine of patients diagnosed with PML between November 2010 and March 2013 was stored at -80°C until further analysis in the virology laboratory of Cochin university hospital. All clinical samples were collected in the frame of standard virology diagnosis procedures, and were independently obtained to confirm JCV infection in immune depressed patients suspected with PML. Samples were stripped of all personal identifiers other than the type of immune depression and the nature of clinical sample. The use of these samples for research (“Analysis of JCV NCCR in PML”) was approved by the Ile de France I Ethics Committee. The characteristics of the 10 PML samples are presented in **S1 Table**.

### JCV viral load

The sequences of all primers and probes used in this study are indicated in **S2 Table**. After DNA extraction using an automated platform (QIAsymphony SP, QIAGEN), JCV genome was amplified by an in house real-time PCR specific for JCV T antigen coding sequence (**S2 Table**). Twenty five µl of TaqMan™ Universal PCR Master Mix (Applied biosystems ™), 4 to 20 ng of DNA and 200 nM (each) of forward primer, reverse primer and probe in a final volume of 50 µl were used to perform the amplification in GeneAmp® PCR System 9700 (Applied Biosystems®) thermocycler. The PCR cycle conditions were as follows: a denaturation/activation step of 10 min at 95°C and then 50 cycles of amplification (95°C for 15 s, 60°C for 1 min). Quantification was obtained by comparison with a standard curve established through 10-fold serial dilution of a standard plasmid (pGem-T-easy containing the JCV T antigen target sequence) covering a dynamic range of 8.10^0^ to 8.10^4^ calculated copy numbers per reaction and yearly verified by participation to quality control for molecular diagnostics assessments.

### NCCR analysis

NCCR sequences were amplified by nested PCR using outer primer pair 1 and inner primer pair 2 (**S2 Table**). HotStartTaq® mix (QIAGEN) was used to amplify 1 to 5 ng of DNA with 400 nM of pair 1 primers using these cycle conditions: a denaturation/activation step of 15 min at 95°C and then 45 cycles of amplification (94°C for 45 s, 55°C for 30 s, 72°C for 45 s). For CSFs from patients 3 and 4, 1 µl of amplicon was used to perform a second PCR with the inner primer pair 2 in the same conditions except that the 35 cycles of amplification were performed with a hybridization step at 52°C. DNA sequencing of PCR products was performed by automatic DNA sequencer (3130 Genetic Analyzer, Applied Biosystems®), according to the manufacturer’s specifications. The sequences were aligned to the CY *at*-NCCR [12] with the program BioEdit and divided into sequence sections *a*, *b*, *c*, *d*, *e*, and *f* consisting of 25, 23, 55, 66, 18 and 69 bp, respectively (**Fig 1**).

### NCCR cloning

NCCR of JCV present in CSF and urine samples of Patient 1 was amplified using specific primers introducing *Afe*I and *Age*I sites in both orientations (**S2 Table**). Each amplification product was cloned into pGem-T-easy (Promega ®) and the generated plasmids were transformed in XL10-Gold® Ultracompetent *E*. *coli* bacteria (Stratagene ®). Bacteria were grown at 37°C in Lennox-Bertani (LB) medium supplemented with ampicillin (100 µg/ml). After cloning into pGem-T-easy, 10 clones from each sample were sequenced in order to verify accuracy of main sequences.

**Fig 2:**
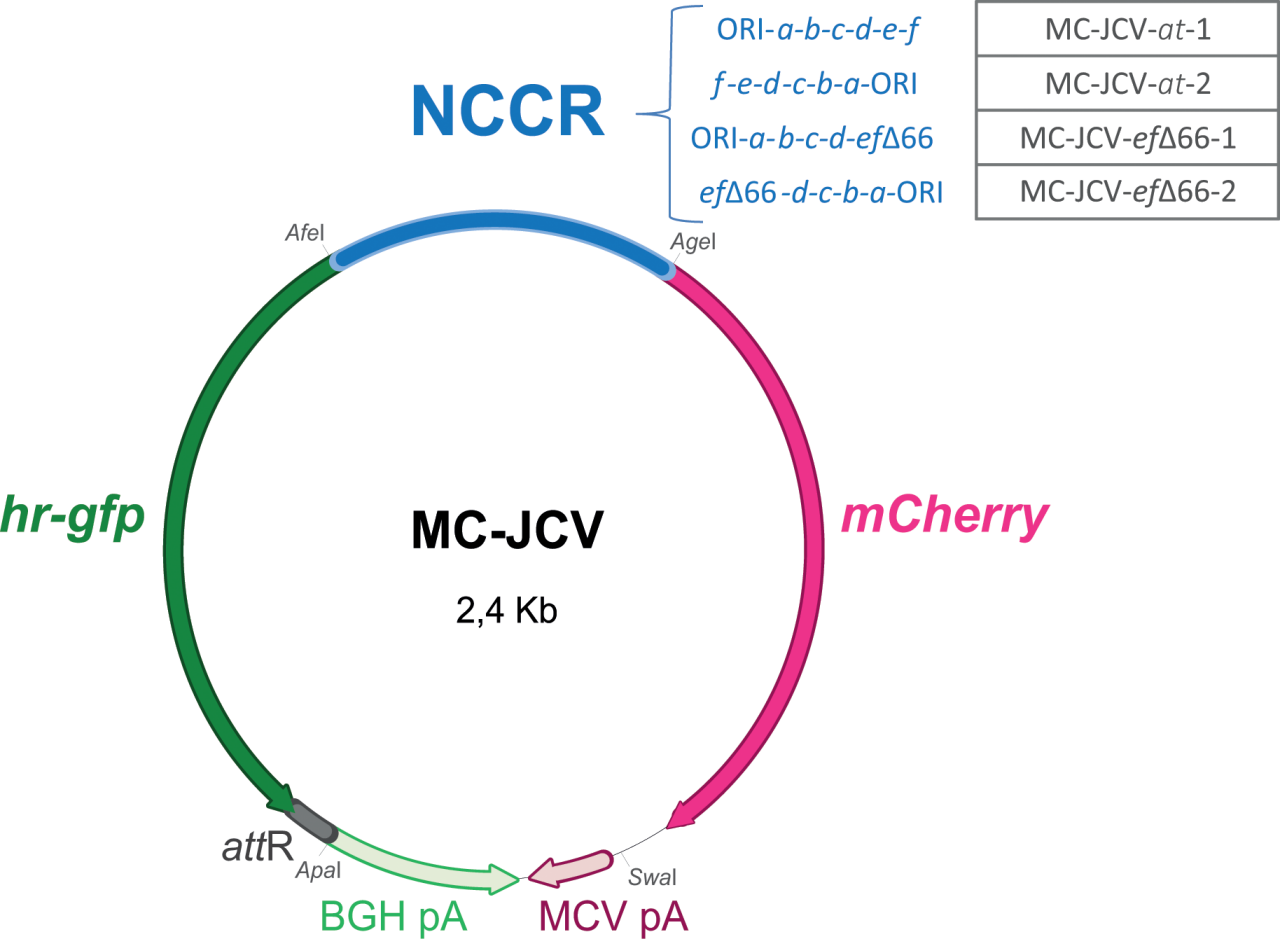
Genetic organization of JCV minicircles. NCCRs were cloned in both orientations in between *hr-gfp* and *mCherry* genes. Each reporter gene is followed by a polyadenylation signal (pA) to ensure transcription termination. *Att*R site corresponds to a scar (≈ 34 bp) remaining after the recombination between the *att*B and *att*P sites present on the parental plasmid. This reaction is catalyzed by PhiC31 integrase and allows the elimination of bacterial plasmid sequences [58]. The name of the constructs used in this study is indicated. The representation is at scale. Other abbreviations: BGH: bovine growth hormone; MCV: Merkel carcinoma cell virus; pA: polyadenylation signal.

The JCV NCCRs (*Age*I-*Afe*I fragments from pGem-T-easy vectors containing JCV NCCRs) were introduced between *Age*I and *Afe*I sites in pMC-JCV plasmid (named “p” for plasmid, and only “MC-JCV” to define to the minicircle form). This plasmid derives from pMC.BESPX-MCS1 (System Biosciences®) and contains in the multiple cloning site: a module (cloned between *Spe*I and *Eco*RV sites) corresponding to *hr-gfp* gene (encoding the humanized renilla green fluorescent protein, hrGFP) followed by an *Afe*I site, an NCCR sequence surrounded by *AfeI* and *AgeI* sites and *mcherry* gene surrounded by *Age*I and *BspE*I sites; the Merkel carcinoma cell virus (MCV) polyadenylation signal (pA) cloned between *Swa*I and *Sac*I sites; and the bovine growth hormone (BGH) pA cloned between *Sac*I and *Apa*I sites, in opposite direction as compared to MCV pA (**Fig 2**). These two polyA signals were introduced to favor the efficient termination of early and late transcription. Distinct polyA sequences were used to limit recombination events between close regions within minicircles. Of note, *hr-gfp* and *mCherry* genes both have the same Kozak sequence controlling the start codon use. All constructs were verified by sequencing.

Two control plasmids were generated from pMC-JCV-*at-*1: one deleted of the *hr-gfp* gene, named “pMC-JCV-mCherry” (obtained by digestion with *Sma*I and *Afe*I, blunting and autoligation of the vector), and the other deleted of the *mCherry* gene, named “pMC-JCV-GFP” (obtained by digestion with *Stu*I and autoligation of the vector) (**Fig 2**).

### Minicircle production

JCV minicircles were produced and purified as recommended by the manufacturer (System Biosciences). Briefly, pMC-JCV plasmids were introduced in ZYCY10P3S2T *E*. *coli* strain (System Biosciences®). For minicircles production, bacteria were grown at 30°C in 200 ml of LB medium supplemented with kanamycin (50 µg/ml). After 16 h, 200 mL of LB medium supplemented with kanamycine (100 µg/mL) and arabinose (2%) were added to the culture. Arabinose induces the expression of PhiC31 integrase and *Sce*-I meganuclease, so that the bacterial sequences of the plasmid are eliminated to produce minicircles. After 5 h at 30°C, bacteria were harvested in order to perform a maxipreparation of DNA following the manufacturer’s recommendations (NucleoBond® Xtra Maxi kit, Malcherey-Nagel). To eliminate traces of genomic DNA, minicircles were purified on 1% agarose gel (Nucleospin® Extract II kit, Macherey-Nagel).

### Cell lines and culture

HEK293H (ThermoFisher Scientific, ref 11631017) and U-87MG (Sigma Aldrich) human cell lines were maintained in Minimum Essential Medium (ThermoFisher Scientific, ref 31095052) supplemented with 10% fetal bovine serum (Eurobio, Dominique Dutscher, ref S1810-100), penicillin G (250 000 UI/L, PANPHARMA, ref 3658208) and gentamicin (40 mg/L, PANPHARMA, 3512025). Of note, HEK293H were established from primary embryonic human kidney and transformed with sheared human adenovirus type 5 DNA. They are devoid of SV40 Large T-antigen (present only in HEK293T derivatives). Cell lines were incubated at 37°C, under an atmosphere containing 5% CO_2_.

### Transfection procedure

The day before transfection, 10^5^ cells per well were seeded in 24-well plates. Two hours before transfection, the culture medium was replaced with fresh medium (supplemented with serum). Cell transfection with 500 ng purified JCV DNA minicircles was performed at 60–80% confluence using 1 µl (to transfect U-87MG cells) or 2 µl (to transfect HEK293H cells) of Lipofectamine ® LTX & PLUS™ Reagent (Invitogen by Life technologies, ref 15338–100) according to the manufacturer’s instructions. The next day, the medium was replaced with fresh medium. Three days after transfection, cells were harvested and fixed with 1% formaldehyde (BD Cytofix™) in phosphate-buffered saline. To limit technical biases, the analysis was performed 10 times independently (at different days) using 3 distinct minicircle preparations. Transfection experiments were performed on U-87MG and HEK293H cell lines in parallel (i.e. the same day with the same minicircle preparations). The plasmid pSYC-97 encoding eGFP-P2A-mCherry (P2A sequence mediating the cleavage between eGFP and mCherry) [49] under the control of a simian cytomegalovirus promoter was used as a positive control to normalize transfection experiments.

### Flow cytometry analysis

Flow cytometry analyses were performed on fixed cells using the BD LSRFortessa™ cytometer (BD Biosciences) and FlowJo software. The hr-GFP fluorescence was excited using a blue laser (488 nm) and detected at 530/30 nm. The mCherry fluorescence was excited using a yellow-green laser (561 nm) and detected at 610/20 nm. Cells harboring a control plasmid devoid of fluorescent genes, pSYC-181 [49], or a plasmid encoding *gfp* only (pMC-JCV-GFP or pIRES-GFP by CLONTECH) or *mCherry* only (pMC-JCV-mCherry or pLeGO-C2 [50]) were employed to calibrate the fluorescence channel voltages and region gates. We used a magnetic gate (FlowJo), centered on each population (≈ 30 000 events), to accurately define gate on population and avoid slight shift between samples.

### Statistical procedure

Transfection experiments were independently repeated at least 10 times. Three independent preparations of each minicircle construct were used. Data were analyzed with R software [51] and statistical significance was assessed by means of Welch two sample *t* tests. Asterisks depict statistical significance (*p<0.05, **p<0.01, ***p<0.001).

## Results & discussion

### Variability of JCV NCCR sequences from PML patients

**Fig 3:**
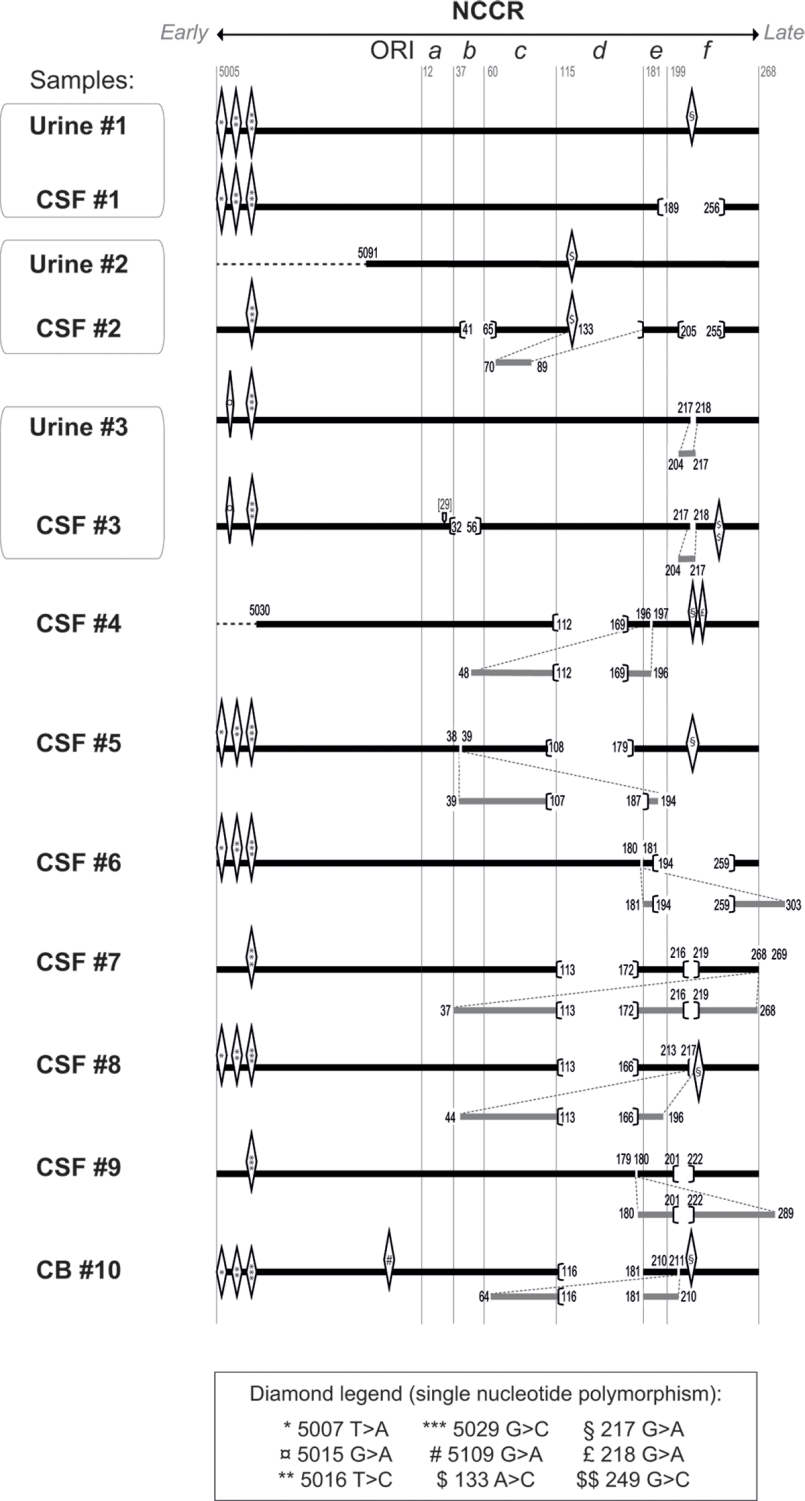
Genetic organization of NCCR sequences obtained from 10 PML patients. NCCR sequences from urine, CSF (cerebrospinal fluid) or CB (cerebral biopsy) samples from 10 patients (from #1 to #10) were aligned against the CY *at*-NCCR. All patients were immune depressed, either due to HIV infection (*n* = 7), Wiskott-Aldrich syndrome (*n* = 1), natalizumab treatment for multiple sclerosis (*n* = 1) or unknown etiology (*n* = 1) (**S1 Table**). Grey numbers indicate the first position of each region in this sequence, as previously described [12]. Point mutations, deletions and rearrangements are represented by diamonds, brackets and grey bars, respectively. Black numbers indicate the position of these mutations, the mentioned position being still present in the NCCR sequence. Dashed black lines represent regions that were not analyzed. Of note, all NCCR from CSFs are of subtype II-R except the ones from CSF#1 (II-S) and from CSF#2 (I-R).

JCV NCCR sequences were obtained from 10 PML independent cases, and in three cases, brain- and urine-derived sequences from the same patient could be compared. The urine-derived sequences were > 99% homologous to published *at-*NCCR (except for a 14 bp nucleotide duplication observed in sample #3), whereas brain-derived sequences greatly varied (**Fig 3**). The region upstream section *a* (including the ORI) was overall highly conserved (**Fig 3**), although point mutations in this region (at positions 5007, 5016 and 5029 as compared to CY *at*-NCCR) were shared between strains from distinct patients, suggesting natural polymorphism of circulating strains. Noticeably, all variations observed within brain-NCCR sections *a* to *f* (9 point mutations, 15 deletions, 10 repeats) differed between patients and from previously described strains. These observations further support the hypothesis that *at* JCV rather than *rr* JCV is involved in inter-individual transmission [11]. Indeed JCV variants may either arise because of i) a loss of selection pressure (out of the kidney compartment) allowing the diversification of JCV NCCRs, or ii) the presence of a tissue-specific pressure within brain that positively selects some JCV variants. In either case, these JCV variants represent an evolutionary dead-end since they do not promote JCV propagation within human populations.

In accordance with NCCR classification, most NCCRs obtained from CSF of PML patients belong to *rr*-NCCRs of subtype II-R (8 out of 10) or to a less extend to subtype I-R (1 out of 10) (**Figs 1**and **3**), as previously described [15, 34, 35, 37, 52] (for review [53]). Interestingly, when a deletion was present within a repeated region (in 7 out of 9 NCCR regions obtained from CSFs containing a repeated region), it was shared by both copies, suggesting that the deletion occurred prior to duplication (**Fig 3**), as previously proposed [34, 54].

Remarkably, CSF sample from a single patient (CSF#1) did not contain a typical *rr*-NCCR JCV. Instead, the NCCR sequence obtained from this sample belonged to type II-S (i.e. archetype-like). This variant, further named *ef*Δ66-NCCR, was atypical because very similar to the archetype *at*-NCCR except for a deletion of 66 bp overlapping *e* and *f* sections.

Interestingly, the *f* region presented at least one deletion or one repeat in 80% of NCCRs from CSFs (**Fig 3**). Although the *f* section was initially excluded from the classification of NCCR variants for purposes of simplification [25], these results suggest that its role in the evolutionary outcome of JCV infection may be underestimated. Indeed, the deletion of the *f* section has been previously described in several patients, mostly combined with duplications [15, 24, 36, 55, 56], and rarely as a single rearrangement [32, 34, 37, 38, 41, 52, 57]. Since NCCRs from urine and CSF samples from Patient 1 were very similar except for the *ef*Δ66 deletion, they represent a good model to investigate the importance of the *f* region in the context of a naturally occurring polymorphism. Moreover, the description of such a II-S variant in CSFs from PML patients is rather atypical and questions its role relative to *at-*NCCR. We thus decided to perform a parallel analysis of gene expression from *at*-NCCR (Urine#1) and *ef*Δ66-NCCR (CSF#1) in cerebral *vs* renal cell types.

### Analysis of the bidirectional NCCR driven-expression of fluorescence reporter genes from minicircles

We therefore developed an original approach to easily monitor the NCCR-driven expression by adapting the emerging technology involving DNA minicircles [58–61] to produce JCV minicircle reporters by recombination (**Fig 2**). This approach is of potential interest in virology since it circumvents the use of constructs containing sequences of bacterial origin. Indeed, these bacterial sequences are likely to lead to biased analysis by modifying the characteristics of epigenetic DNA in eukaryotic cells [58, 62]. Fluorescent mCherry and hrGFP encoding reporter genes were cloned under the control of JCV NCCRs from Patient 1, in sense or antisense orientation. This way, four plasmids were generated, namely: pMC-JCV-*at-*1 (containing *at*-NCCR which early orientation controls *gfp* expression), pMC-JCV-*at-*2 (containing *at*-NCCR which early orientation controls *mCherry* expression), pMC-JCV-*ef*Δ66–1 (containing a NCCR variant with a 66 bp deletion overlapping *e* and *f* regions, and which early orientation controls *gfp* expression), pMC-JCV-*ef*Δ66–2 (containing a NCCR variant with a 66 bp deletion overlapping *e* and *f* regions, and which early orientation controls *mCherry* expression) (**Fig 2**). Each reporter gene is followed by a specific pA signal to ensure transcription termination.

We explored JCV minicircle expression in two human cell lines chosen to represent renal (HEK293H, devoid of T-antigens) and glial (U-87MG) contexts, the latter previously used to study JCV expression [37, 45–47]. Only few studies compared glial versus renal cell lines, using mostly tumor derived cell lines [37, 45, 63] which are more convenient for a routine reporter assay than human primary cells [64]. These experiments were performed in absence of polyomavirus T-antigens, so that our experimental model strictly reflects the basal promoter activity of JCV NCCRs (independently of JCV replication).

**Fig 4:**
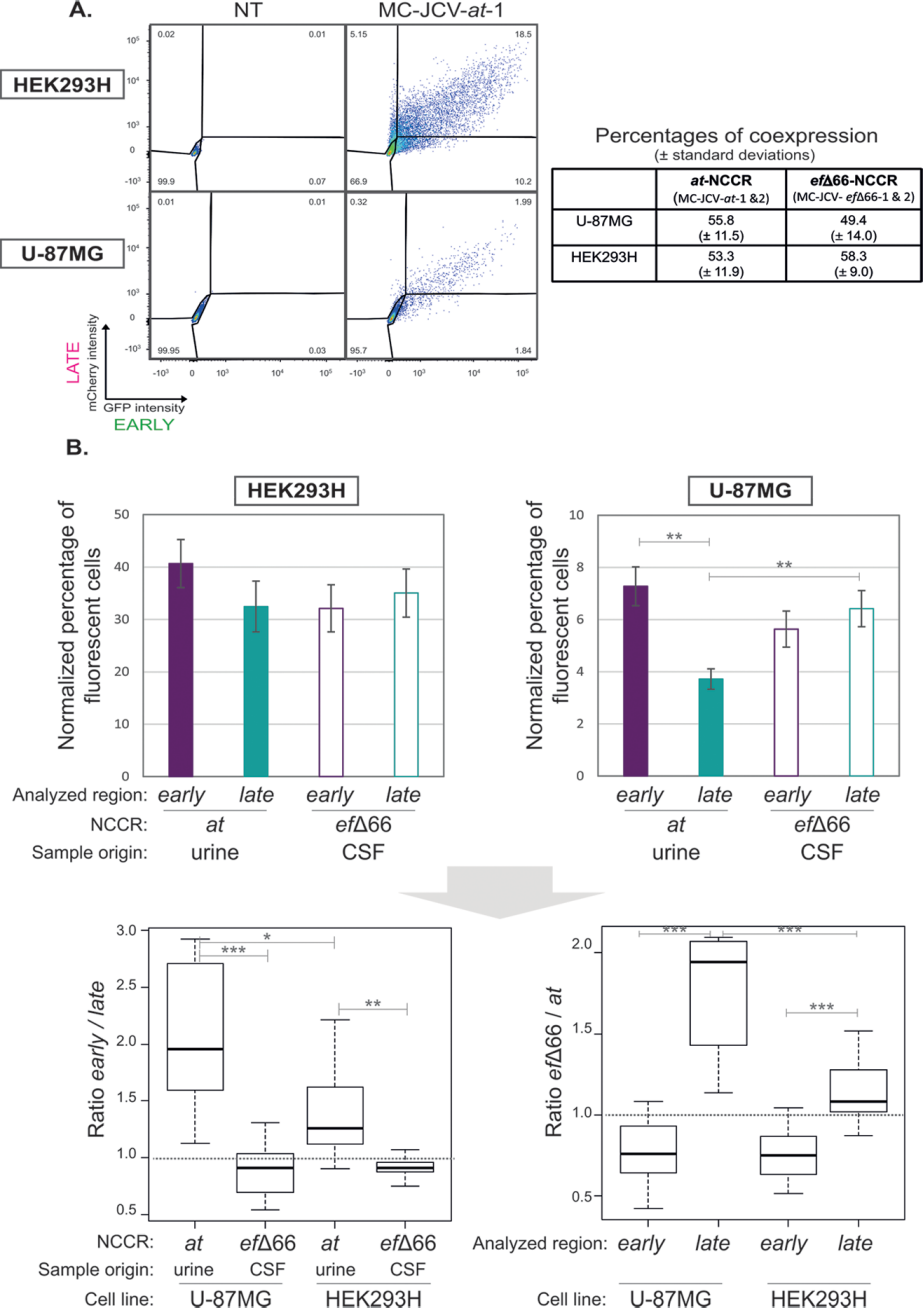
Fluorescent reporter expression from JCV minicircles. **(A) Pattern of fluorescent gene expression from JCV minicircles in HEK293H and U87-MG cell lines.** Cell fluorescence was measured by cytometry analysis 3 days post-transfection with MC-JCV-*at*-1. One representative experiment (over 10 independent experiments) is presented. The efficiency of transfection, assessed with pSYC-97 plasmid, was of 99% and 82% for HEK293H and U87-MG cells, respectively. Three populations of fluorescent cells were observed: GFP^+^mCherry^-^, GFP^+^mCherry^+^ and GFP^-^mCherry^+^. Similar patterns of expression were obtained with other JCV minicircles (MC-JCV-*at*-2, MC-JCV-*ef*Δ66–1, MC-JCV-*ef*Δ66–2). The percentages of cells coexpressing early and late regions within the whole population of fluorescent cells (expressing *gfp* and/or *mCherry* reporter genes) after transfection of JCV minicircles are indicated (means ± standard deviations of values obtained during 10 independent experiments performed with *at-* or *ef*Δ66-NCCR minicircles in both orientations). NT: not transfected. **(B) Reporter gene expression depending on cell- and NCCR- type.** Cell fluorescence was assessed by cytometry analysis 3 days post-transfection of HEK293H or U-87MG with MC-JCV-*at*-1, MC-JCV-*at*-2, MC-JCV-*ef*Δ66–1 or MC-JCV- *ef*Δ66–2. The percentages of fluorescent cells (expressing mCherry at detectable level) were normalized on transfection efficiency using pSYC97 as a positive control of transfection. Values are means ± standard errors from 10 independent experiments. They reflect either the NCCR-driven expression in the late (MC-JCV-*at*-1, MC-JCV-*ef*Δ66–1) or early (MC-JCV-*at*-2, MC-JCV- *ef*Δ66–2) orientation. The ratios of these percentages are also represented. Statistical differences are indicated (see M&M). CSF: cerebrospinal fluid.

Three days post transfection by JCV minicircles or control plasmids, green and red fluorescences were assessed by cytometry analysis. In order to avoid any bias linked to differences in transfection efficiency between HEK293H and U-87MG cells, we used a control plasmid to normalize the percentage of fluorescent cells obtained after JCV minicircles transfection on the percentage of fluorescent cells measured with the control plasmid. This way, the experiments were normalized regarding transfection efficiency using pSYC-97 (containing *gfp-P2A-mCherry* under the control of a simian CMV) [49] as a positive control since its transfection leads to the highest percentages of fluorescent cells in both cell lines (as compared to other positive controls used to calibrate the fluorescence channel voltages and region gates such as pIRES-GFP or pLeGO-C2 [50]). HEK293H and U-87MG cells were efficiently transfected in these experimental conditions, although lower percentages of transfection of pSYC-97 were generally obtained in U-87MG cell line (on average 98.1 ± 2.5 and 66.8 ± 15.9% of transfection, respectively–*n* = 10). The percentages of fluorescent cells obtained after transfection with pSYC-97 were higher than with MC-JCVs, suggesting that JCV NCCRs are almost 2.5 to 10 fold weaker than simian CMV promoter in HEK293H and U-87MG cell lines, respectively (**Fig 4**).

Interestingly, JCV NCCR-driven expression was quite heterogeneous within both cell lines. Firstly, GFP and mCherry fluorescence levels varied on a 5 log_10_ dynamic range in each cell line (**Fig 4A**). Secondly we observed 3 sub-populations of cells expressing transgene from JCV NCCR at detectable levels: early only-, late only-, and early and late-expressing cells, this latter being dominant (**Fig 4A**). This suggests that although the polyomaviral cycle was initially described as hierarchically ordered in two sequential early and late phases separated by DNA replication [21–23, 65], JCV NCCR-driven expression may be less deterministic than expected. Instead JCV expression could be stochastic at the initial expression steps, the expression of the « late » region being possibly early and effective even in absence of T proteins. These results are in accordance with previous studies reporting that early and late expressions of polyomaviruses can be detected simultaneously in a population of cells [23, 37, 66–69]. Here we observed that the JCV NCCR-driven expression of early and late regions can occur concomitantly at the single cell level, suggesting that, in our experimental conditions, a significant part of the cell population is competent for NCCR bidirectional promoter activities (from the same and/or distinct DNA templates).

### A deletion within *e* and *f* sections restores NCCR-driven expression in glial cells

We therefore performed a comparative analysis of NCCR-driven expression (normalized on pSYC97 expression) using the same reporter as a reference, to avoid bias due to difference in fluorescent protein expression, stability and/or detection. The mCherry was chosen since it was slightly better expressed (and/or detected) than GFP in both cell lines.

First, we observed that both *at*- and *ef*Δ66-NCCR driven expressions were 6 to 9 fold lower in U-87MG than in HEK293H cells (*p*<0.001 both for early and late regions, in both type of NCCR) (**Fig 4B**), indicating that U-87MG cells are less permissive to JCV expression than HEK293H cells. Second, the *at*-NCCR driven expression was significantly higher in the early orientation than in the late one in U-87MG (2.1 fold decrease, *p*<0.001) (**Fig 4B**). This indicates that the late expression from *at-*NCCR is particularly disfavoured in this glioblastoma cell line, accordingly to previous studies [46, 47]. This might promote the selection of variants *in vivo*. Interestingly, the *ef*Δ66-NCCR driven late expression was similar to early expression in both HEK293 and U-87MG cells (**Fig 4B**). This indicates that the *ef*Δ66 deletion restored late expression in the glioblastoma cell line with no impact on late expression in HEK293H cells. In parallel, this mutation has no significant impact on the early expression regardless of the cell type (**Fig 4B**), emphasizing that the potential gain associated with the *ef*Δ66 deletion only concerns late expression. These results illustrate how the loss of sequences can increase viral expression, a situation analogous to the “black holes” described for bacterial pathogenicity [70]. The 66 bp sequence absent within the *ef*Δ66-NCCR is very close to the late region and contains binding sites for Spi-B [71], NFI factors [72] and AP1 [73] transcription factors. Further investigations will be required to understand how their deletion can increase late JCV expression. We hypothesize that some transcription factors recognizing the *f* section repress JCV expression as previously described in the case of NFI-A [74] and AP1 [73].

## Conclusion

In conclusion we evidenced a relevant link between one JCV NCCR natural polymorphism and cell-type dependent expression using minicircles. Altogether our results suggest that the archetype NCCR-driven late expression may be a limiting factor in glial cells. This study opens new insights for monitoring the impact of NCCR variation on JCV early and late region expression. The JCV minicircles developed in this study could be adapted to explore the role of other determinants of JCV expression such as notably the native overlapping termination region or the T antigens.

## Supporting information

S1 Table Characteristics of PML samples. (DOCX)

S2 Table Primers and probe used in this study. (DOCX)
