# Supplementary material for: Exploring the role of NCCR variation on JC polyomavirus expression from dual reporter minicircles
Source: PLoS One. 2018 Jun 26;13(6):e0199171. doi: 10.1371/journal.pone.0199171 (PMC6019678; doi:10.1371/journal.pone.0199171)
Supplement: S1 Table — (DOCX) [file pone.0199171.s001.docx]

**Supporting information S1 Table: Characteristics of PML samples**

| **Patient #** | **Underlying immune deficiency^a^** | **Sample^b^** | **JCV viral load (log copy/ml)** |
| --- | --- | --- | --- |
| 1 | HIV | CSF | 4.7 |
|  |  | Urine | 6.9 |
| 2 | HIV | CSF | 4.5 |
|  |  | Urine | 7.5 |
| 3 | HIV | CSF | 2.3 |
|  |  | Urine | 4.7 |
| 4 | HIV | CSF | 4.4 |
| 5 | WAS | CSF | 4.1 |
| 6 | HIV | CSF | 2.5 |
| 7 | HIV | CSF | 5.1 |
| 8 | NA | CSF | 3.1 |
| 9 | HIV | CSF | 6.0 |
| 10 | MS | Cerebral biospy | NA |

^a^HIV (human immunodeficiency virus), MS: multiple sclerosis (natalizumab), WAS: Wiskott-Aldrich syndrome, NA: Not available

^b^CSF: cerebrospinal fluid; NA: Not available
