# Supplementary material for: Exploring the role of NCCR variation on JC polyomavirus expression from dual reporter minicircles
Source: PLoS One. 2018 Jun 26;13(6):e0199171. doi: 10.1371/journal.pone.0199171 (PMC6019678; doi:10.1371/journal.pone.0199171)
Supplement: S2 Table — (DOCX) [file pone.0199171.s002.docx]

**Supporting information S2 Table: Primers and probe used in this study**

| **Primer and/or probe names** | **Sequence (5’ – 3’)*** |
| --- | --- |
| **Determination of JCV viral load by qPCR** | |
| T-antigen-forward | CTTTTTAGGTGGGGTAGAGTGTTG |
| T-antigen-reverse | TCCTGGTGGAATACATTTAATGAGAAG |
| 6-carboxyfluorescein-labeled  T-antigen probe | **FAM**-CATGGCAAAACAGGTCT-**MGB** |
| **NCCR amplification for sequencing** | |
| outer primer pair 1-forward | GATTCCTCCCTATTCAGCACTTTG |
| outer primer pair 1-reverse | CACCTGTGCAAAAGTCCAGC |
| inner primer pair 2 - forward | GGCCTCCTAAAAAGCC |
| inner primer pair 2 - reverse | TCCACTCCAGGTTTTACTAA |
| **NCCR cloning** | |
| CSF sample GFP Early - mCherry Late - *Afe*I-forward | agcgctTTTAGCTTTTTGCAGC |
| CSF sample GFP Early - mCherry Late – *Age*I - reverse | accggtGGCCAGCTGGTGTTGTTTAC |
| CSF sample mCherry Early - GFP Late - *Age*I - forward | accggtTTTAGCTTTTTGCAGC |
| CSF sample mCherry Early - GFP Late – *Afe*I - reverse | agcgctGGCCAGCTGGTGTTGTTTAC |
| Urine sample GFP Early - mCherry Late – *Afe*I - forward | agcgctTTTAGCTTTTTGCAGC |
| Urine sample GFP Early - mCherry Late - *Age*I - reverse | accggtGGCCAGCTGGTGACAAGCC |
| Urine sample mCherry Early - GFP Late - *Age*I - forward | accggtTTTAGCTTTTTGCAGC |
| Urine sample mCherry Early - GFP Late – *Afe*I - reverse | agcgctGGCCAGCTGGTGACAAGCC |

* Restriction sites are written in lowercase
